# The Interrelated Effect of Cations and Electrolyte pH on the Hydrogen Evolution Reaction on Gold Electrodes in Alkaline Media

**DOI:** 10.1002/anie.202102803

**Published:** 2021-05-07

**Authors:** Akansha Goyal, Marc T. M. Koper

**Affiliations:** ^1^ Leiden Institute of Chemistry Leiden University PO Box 9502 2300 RA Leiden The Netherlands

**Keywords:** electrocatalysis, gold, hydrogen evolution reaction

## Abstract

In this work we study the role of alkali metal cation concentration and electrolyte pH in altering the kinetics of the hydrogen evolution reaction (HER) at gold (Au) electrodes. We show that at moderately alkaline pH (pH 11), increasing the cation concentration significantly enhances the HER activity on Au electrodes (with a reaction order ≈0.5). Based on these results we suggest that cations play a central role in stabilizing the transition state of the rate‐determining Volmer step by favorably interacting with the dissociating water molecule (*H–OH^δ−^–cat^+^). Moreover, we show that increasing electrolyte pH (pH 10 to pH 13) tunes the local field strength, which in turn indirectly enhances the activity of HER by tuning the near‐surface cation concentration. Interestingly, a too high near‐surface cation concentration (at high pH and high cation concentration) leads to a lowering of the HER activity, which we ascribe to a blockage of the surface by near‐surface cations.

## Graphical abstract

The kinetics of the hydrogen evolution reaction (HER) at gold electrodes is affected by the concentration of alkali metal cations and electrolyte pH. At moderately alkaline pH, increasing the cation concentration greatly enhances the HER activity. Cations are proposed to help stabilize the transition state of the rate‐determining Volmer step by a favorable interaction with the dissociating water molecule (*H–OH^δ−^–cat^+^).

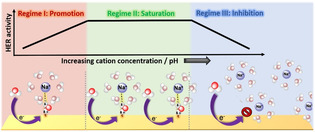

## Introduction

Research on the electrochemical hydrogen evolution reaction (HER) is at the heart of realizing a sustainable and economically feasible hydrogen‐based economy. Additionally, this “simple” two‐electron transfer reaction serves as a test ground for the laws of electrocatalysis and therefore continues to be of utmost importance, both in fundamental electrochemistry and for application purposes. However, the rigorous experimental and theoretical studies that have been undertaken to discern the activity descriptors governing the kinetics of HER at acidic pH (2 H^+^ + 2 e^−^ → H_2_; proton reduction),[^1a^, ^1b^, ^1c^] cannot satisfactorily explain the activity trends that have been observed at alkaline pH (2 H_2_O + 2 e^−^ → H_2_ + 2 OH^−^; water reduction).^[2]^ This gap in the understanding of HER in alkaline media is a major hindrance in the optimization of alkaline water electrolysers, which can in principle be more cost efficient than the acidic Proton Exchange Membrane electrolysers.^[3]^

The major caveat in the present understanding of HER in alkaline media arises from two main factors, (1) lack of systematic studies on surfaces other than Pt in a broad pH window,^[4]^ and (2) sole focus on the hydrogen adsorbed on the metal surface (H_upd_ and H_opd_) as the key descriptor for HER.^[5]^ Platinum is considered the best metal for HER since it catalyzes HER at negligible overpotential (in acidic media), owing to its optimal hydrogen binding energy (Δ*G*_H,adsorption_≈0). The large variations in the rate of HER (up to few orders of magnitude) on different electrode materials have been typically correlated to the variations in the free energy of hydrogen adsorption on these catalysts.[^1a^, ^5b^, ^5c^] It is reasonable to assume that these activity trends would also hold in alkaline pH. However, there is ample experimental evidence that catalysts that bind hydrogen less optimally than Pt (such as Pt‐Ru alloys, Ir and 3d metal hydroxide, chalcogenide and phosphide modified electrodes) show superior catalytic activity in alkaline media.^[6]^ Additionally, the loss in the activity of HER on the different crystal facets of Pt in going from acidic pH to alkaline pH, cannot be explained satisfactorily by the changes in the hydrogen binding energy (HBE) either. Yan and co‐workers have suggested that the sluggish kinetics of HER in alkaline media can be attributed to the increasing HBE with increasing pH, as derived from the positive shift of the underpotential hydrogen (H_upd_) peak in the blank voltammetry.^[5a]^ However, it has been shown that the positive shift in the H_upd_ peak arises from the weakening of the OH adsorption on Pt(100) and Pt(110) sites due to the presence of alkali metal cations near the interface, and are not due to the changes in the HBE.^[7]^ Moreover, unlike Pt(100) and Pt(110), Pt(111) does not show changes in the experimentally observed HBE with a change in electrolyte pH, however it still shows a drastic drop in the HER activity as the electrolyte pH is increased.^[8]^ These apparently conflicting trends point to a fundamentally different nature of HER in alkaline media, where the dissociation of water at the metal interface can introduce an additional energy barrier for the reaction and therefore, the overall reaction rate can depend on additional factors, such as the interaction of water and its dissociation products with the (electro‐)chemical environment at the metal‐electrolyte interface. Recently, our group has shown that reorganization of interfacial water may be an important descriptor for the activity of HER in alkaline media on Pt(111), which can be modified indirectly via the electrolyte pH and/or by the clusters of Ni(OH)_2_ at the surface through their influence on the potential of zero charge and the resulting interfacial electric field. These results showed that the interfacial electric field affects the structure of the water network at the interface which in turn controls the HER kinetics in alkaline media.^[8]^ Nevertheless, in order to arrive at a clear molecular picture of what dictates the activity of HER in alkaline media, it is vital to also probe the short‐range interactions that can affect the metal‐water interface locally.

In this regard, the non‐covalent interactions between the water molecules and alkali metal cations have been shown to play a significant role in determining the HER activity by locally interacting with the reactants/products of HER. Most notably, Markovic and co‐workers have probed the promotional role of Li^+^ ions in improving the HER activity in alkaline media.[^6a^, ^9^] More recently, Grimaud and co‐workers have shown that these effects are also operational in organic electrolytes.^[10]^ In general, these studies attribute the promotion of the electrochemical water dissociation step to favorable cation‐water interactions. However, various discrepancies still exist in the current literature since the HER activity has been observed to decrease from Li^+^ to Cs^+^ on the different facets of Pt and Ir, while the opposite trend has been observed on Au and Ag.^[11]^ The trend on Pt and Ir is in good agreement with the previous works of Markovic and co‐workers, however, the discriminant behavior of the alkali metal cations on the transition metal electrodes (Pt, Ir and Rh) and the coinage metal electrodes (Au and Ag) indicates that a wide range of electrode‐electrolyte combinations needs to be probed in order to completely understand the role of metal‐adsorbate interactions in the kinetics of HER in alkaline media.

In this work, we address these issues by systematically studying HER in alkaline media on Au electrodes. We will show that the HER activity on polycrystalline Au and Au(111) surfaces is enhanced significantly with the increasing alkali metal cation concentration in the electrolyte, but only in a limited pH region around pH 11. We propose that the cations near the interface interact favorably with the transition state of the rate‐determining Volmer step by stabilizing the (partially) negative hydroxide which is being split off from the reacting water molecule (*H–OH^δ−^–cat^+^). Remarkably, at higher pH, the effect of the concentration of alkali cations is diminished, and it is even negative at pH 13. Furthermore, capacitance curves obtained from impedance spectroscopy suggest that the electrolyte pH also influences the near surface composition of the electrolyte such that an increasing electrolyte pH leads to a corresponding increase in the near‐surface cation concentration. This results in an apparent pH dependence for the HER activity on the Au electrodes where similar to the cation concentration effect, saturation is observed at extreme pH values (pH 13 to pH 14). We attribute the saturation and inhibitive effects observed at high pH and at high cation concentration to a blockage of the surface by cations when they reach a threshold concentration.

This work shows that the electrolyte pH and the near‐surface cation concentration are inter‐dependent parameters, which cannot be easily de‐coupled in alkaline media. Hence, our work provides foundational insights on the complex molecular origin of the pH dependence of HER, and we believe that these insights will be instrumental in guiding further fundamental work and eventually the design of optimized catalyst‐electrolyte conditions for HER in alkaline media.

## Results

### Role of Cations in the HER Kinetics in Alkaline Media

**Figure 1:**
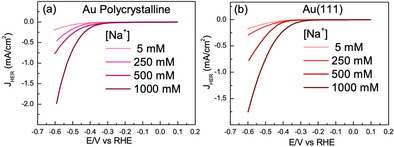
Cyclic voltammograms obtained for HER on a) Au polycrystalline surface and b) Au(111) surface at 2500 rpm in 0.001 M NaOH (pH 11) for different concentrations of NaClO_4_ (5 mM, 250 mM, 500 mM and 1000 mM) in Ar‐saturated environment at 25 mV s^−1^.

**Figure 2:**
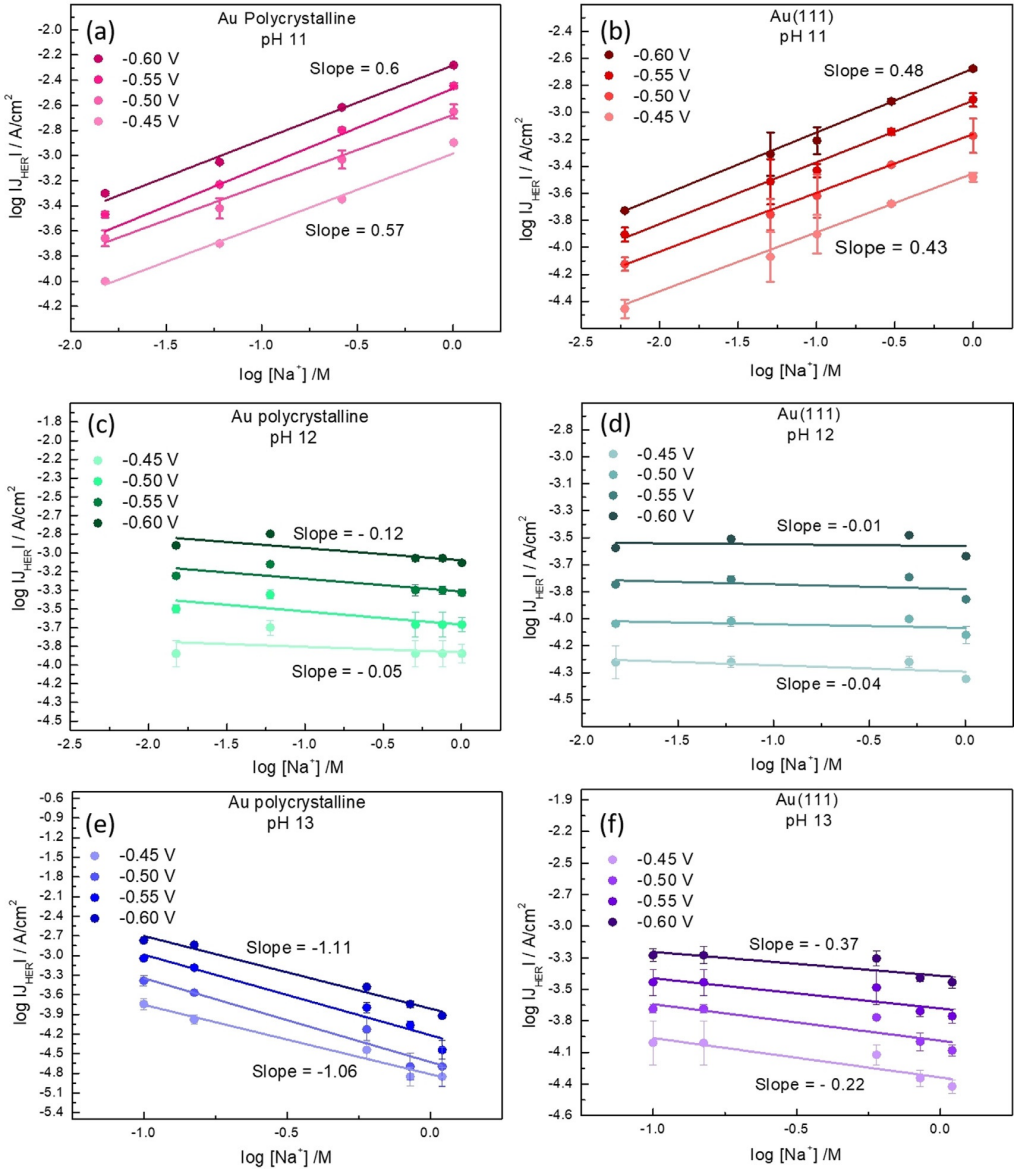
Reaction order plot of HER in the cation concentration at pH 11 on a) Au polycrystalline and b) Au(111), at pH 12 on b) Au polycrystalline and d) Au(111) and at pH 13 on e) Au polycrystalline and f) Au(111), at 50 mV potential steps (vs. RHE) plotted as a function of the logarithm of the current density on the *y*‐axis and logarithm of the [Na^+^] concentration on the *x*‐axis. The corresponding slopes (reaction orders) are indicated next to the plots, where the slope at the bottom corresponds to the applied potential of −0.45 V (vs. RHE) and the slope at the top corresponds to the applied potential of −0.60 V (vs. RHE) in all the graphs.

First, we examine the effect of cation concentration on the kinetics of HER, for constant values of the electrolyte pH. In Figure 1 a and b we show that at moderately alkaline electrolyte pH (pH 11), the HER activity increases significantly with increasing Na^+^ cation concentration in the electrolyte, both on polycrystalline Au and Au(111) surfaces. These experiments illustrate that on a Au electrode, at pH 11, increasing the (near‐surface) concentration of the cations positively affects the kinetics of HER in the alkaline media. Interestingly, measurements at higher pH (shown in Figure 2) show that HER reaction orders in cation concentration are pH dependent. The HER reaction order in cation concentration is around 0.5 at pH 11 (shown in Figure 2 a and b), around 0 at pH 12 (shown in Figure 2 c and d), whereas at pH 13 negative reaction orders are obtained (shown in Figure 2 e and f). We note that for isolated data points in Figure 1 (especially pH 11 and 5 mM NaClO_4_), we cannot neglect the possible contribution of OH^−^ migration to the measured current. However, this effect can be safely neglected for the higher concentrations of NaClO_4_, and therefore there is no significant effect of OH^−^ migration on the derived reaction orders. The pH dependence of the (fractional) reaction orders suggests that the cation induced alteration in the rate limiting step affects a species which is adsorbed at the electrified interface. The Tafel slope of ≈120 mV dec^−1^ in the low overpotential range (see Figure S2 in the Supporting Information) further indicates that the first electron transfer step (H_2_O + e^−^ + * → H−* + OH^−^; Volmer step) is rate determining.^[12]^ In this scenario, fractional reaction orders at pH 11 correspond to a regime with intermediate cation concentration in the double layer which will in‐turn lead to an intermediate coverage of the activated water molecule at the interface.

The near zero reaction orders obtained at pH 12 indicate that, in addition to the bulk cation concentration, the electrolyte pH also affects the near‐surface concentration of cations. A pH dependent cation concentration near the interface can be rationalized based on the fact that the potential of zero charge (*E*_pzc_) shifts positively with the increasing electrolyte pH (*E*_pzc_=*E* ^o^_pzc_ + 0.059 pH; *E*_pzc_=1.12 V vs. RHE for Au(111) at pH 11), thus resulting in a quite negative interfacial electric field (Δ*E*=*E*−*E*_pzc_) under the conditions used in these measurements.^[13]^ Hence, it can be expected that at these moderately alkaline conditions the near surface cation concentration starts to approach saturation.

Interestingly, a closer look at the effect of the cation concentration changes at pH 12 (see Figures 2 c and d and Figure S3 in Supporting Information) reveals that while an initial increase in the cation concentration shows a small positive effect on the activity of HER, at higher concentrations, a slight drop in the HER activity is observed. Furthermore, at pH 13 the increasing cation concentration exhibits an entirely inhibitive effect on HER activity (see Figures 2 e and f and Figure S3 in Supporting Information). These results show that above a certain threshold concentration, the promotional effect of the cations first plateaus and then inhibits the kinetics of HER. Here, the pH dependence of HER reaction orders in cation concentration would signify a correlation between the electrolyte pH and the near‐surface cation concentration. In the next section we elucidate these effects further by studying the role of electrolyte pH in tuning the kinetics of HER for a constant value of bulk cation concentration.

### HER Kinetics in Alkaline Media as a Function of the Bulk pH

**Figure 3:**
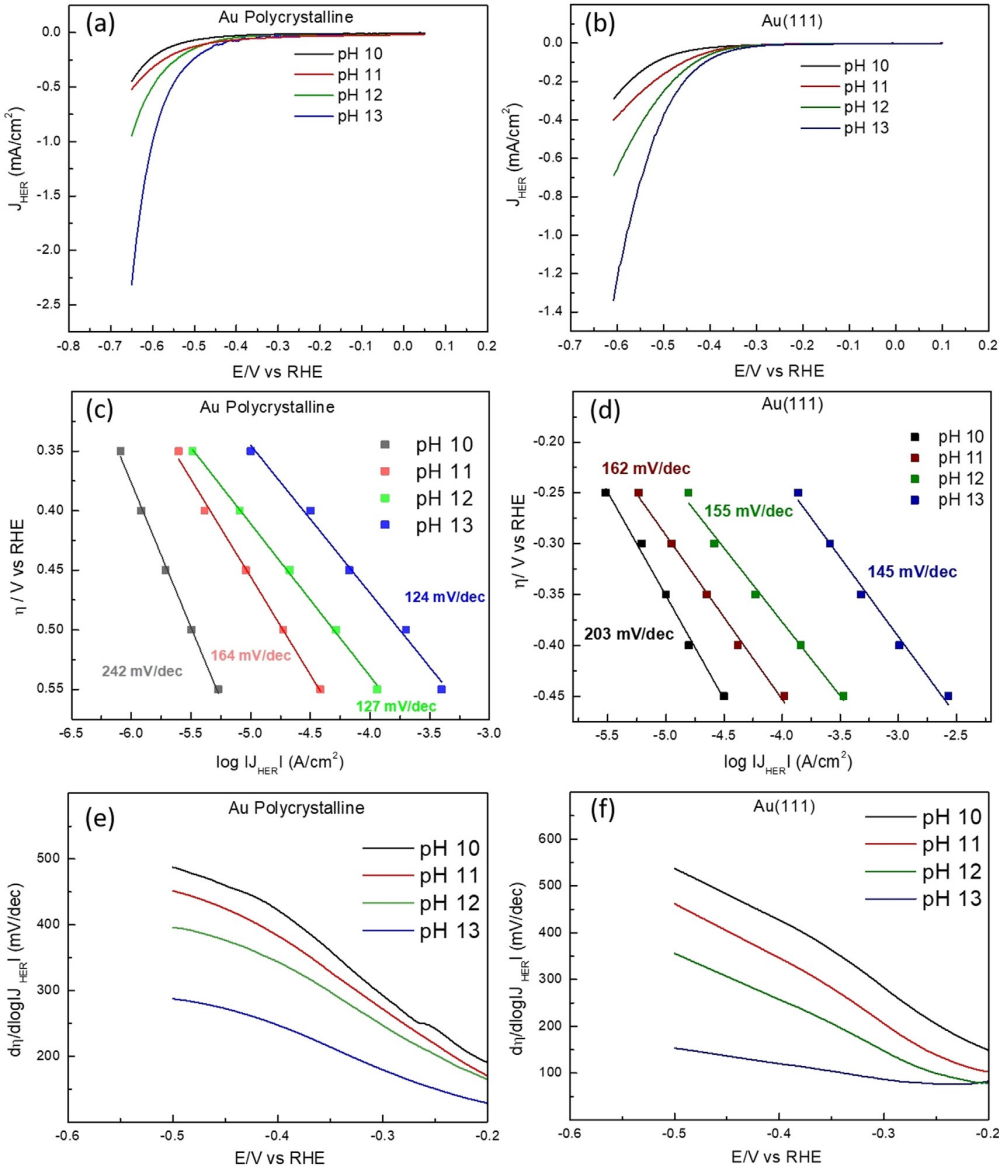
Cyclic voltammograms obtained for HER on a) Au polycrystalline surface and b) Au(111) surface at 2500 rpm in 0.1 M NaOH (pH 13), 0.01 M NaOH +0.09 M NaClO_4_ (pH 12), 0.001 M NaOH + 0.099 M NaClO_4_ (pH 11), and 0.0001 M NaOH +0.0999 M NaClO_4_ (pH 10), in Ar‐saturated environment at 25 mV s^−1^. Steady‐state current obtained at 50 mV potential steps at 2500 rpm on c) Au polycrystalline surface and d) Au(111) surface at different pH values (same as above), plotted as function of the applied overpotential on the *y*‐axis and the logarithm of the current density on the *x*‐axis where the corresponding Tafel slopes at each pH value are indicated next to the plot. Tafel slopes obtained from the differentiation of the cyclic voltammograms in (a) and (b) plotted as a function of the applied potential (vs. RHE) for e) Au polycrystalline and f) Au (111) at different pH values.

In Figure 3 a,b we show that both polycrystalline Au and Au(111) exhibit an increase in the HER activity on the RHE scale with increasing electrolyte pH, at a constant concentration of cations (0.1 M) in the bulk. Moreover, chronoamperometry measurements in Figures 3 c and d show that the steady‐state currents for the HER also increase with the increasing pH. The Tafel slopes thus obtained decrease with the increasing pH, confirming that the increasing electrolyte pH enhances the potential dependence of the HER reaction on Au electrodes. Additionally, in Figures 3 e and f we plot the Tafel slopes, as derived from the cyclic voltammograms, as a function of the applied potential, confirming the trend of the steady‐state chronoamperometry: Tafel slopes of around 120 mV dec^−1^ are obtained at low overpotentials for all the pH values and they increase with lower pH.

**Figure 4:**
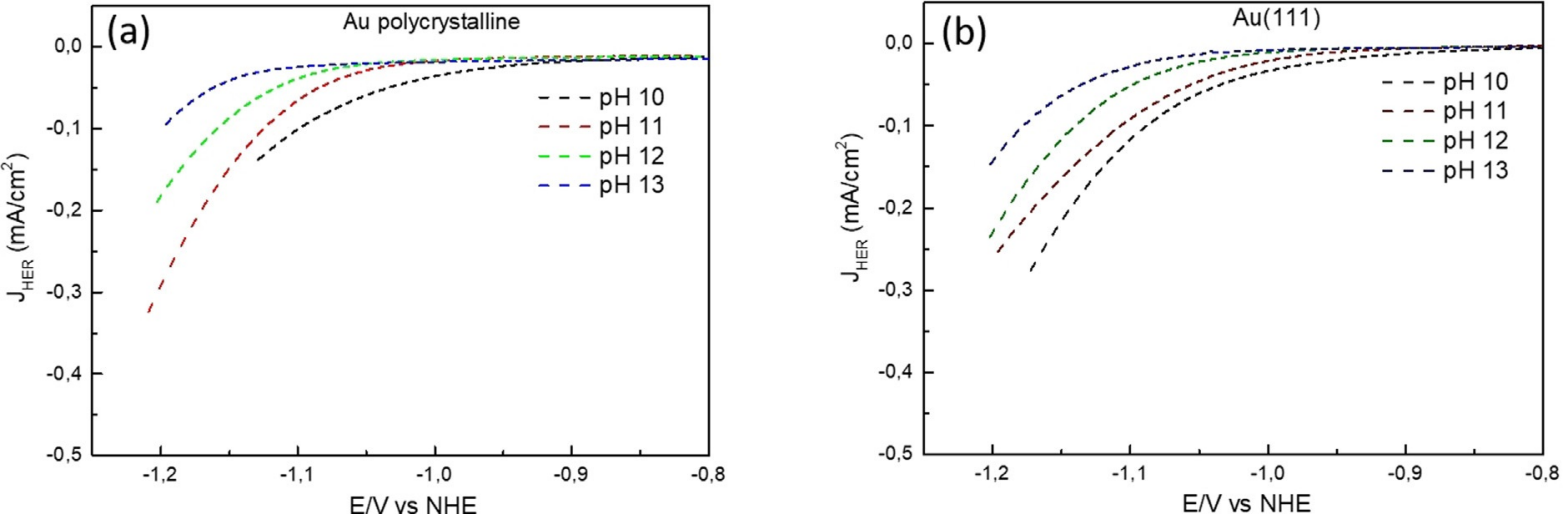
Cyclic voltammograms for HER plotted on the NHE scale for a) Au polycrystalline surface and b) Au(111) surface at 2500 rpm in 0.1 M NaOH (pH 13), 0.01 M NaOH +0.09 M NaClO_4_ (pH 12), 0.001 M NaOH + 0.099 M NaClO_4_ (pH 11), and 0.0001 M NaOH +0.0999 M NaClO_4_ (pH 10), in Ar‐saturated environment at 25 mV s^−1^ where the data has been obtained from Figure 3 a,b, respectively, by converting the potentials from the RHE scale to the NHE scale (*E*_NHE_=*E*_RHE_−0.059pH).

The pH dependence of the HER kinetics is interesting because thermodynamically, the onset for HER is expected to remain constant on the pH dependent RHE scale (*E*_RHE_=*E*_NHE_ + 0.059 pH), because for a given potential on the RHE scale, the thermodynamic driving force is the same, regardless of pH. However, if the Volmer step is indeed rate limiting (as indicated by the Tafel slopes), the kinetics for this reaction should not depend on the electrolyte pH because no proton or hydroxide is involved in the reactant side of the rate limiting reaction equation (H_2_O + e^−^ + * → H−* + OH^−^), implying that the rate should be constant on the pH independent NHE (Normal Hydrogen Electrode) reference scale. Figure 4 shows the data of Figures 3 a and b on the NHE scale. Remarkably, there is also a pH dependence of the kinetics of HER under alkaline conditions on the NHE scale: the HER kinetics become slower with increasing pH, in contrast to the situation on the RHE scale, where the reaction becomes faster. Since the (bulk) cation concentration is constant in these measurements, this result appears to imply an intrinsic pH dependence of the HER on Au.

**Figure 5:**
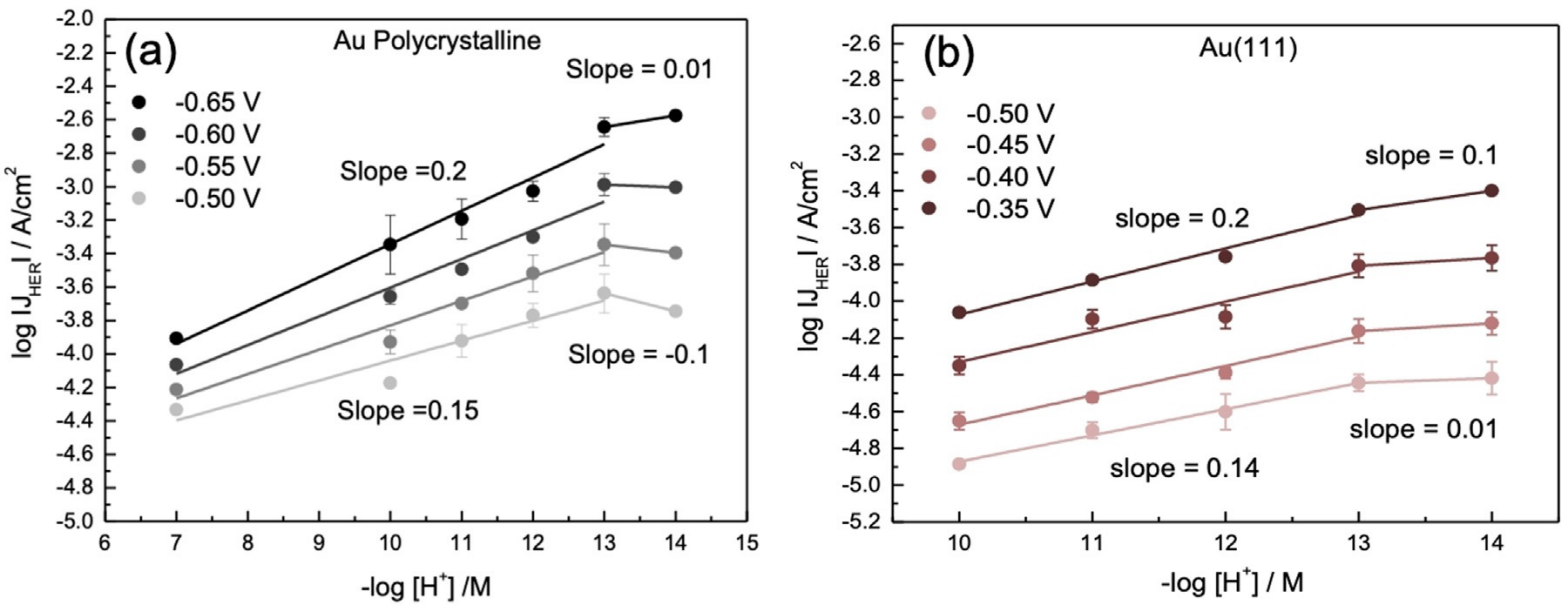
Reaction order of HER in bulk electrolyte pH for a constant concentration of cations in the bulk (0.1 M; except pH 14 where it is 1 M) on a) Au polycrystalline and b) Au(111) at 50 mV potential steps (vs. RHE) plotted as a function of the logarithm of the current density on the *y*‐axis and bulk pH on the *x*‐axis. The corresponding slopes (reaction orders) are indicated next to the plots, where the slope at the bottom corresponds to the applied potential of −0.50 V (vs. RHE) and the slope at the top corresponds to the applied potential of −0.65 V (vs. RHE).

The enhancement in the HER kinetics with the increasing electrolyte pH (on the RHE scale) agrees with the observed pH dependence of cation concentration effects. Together, these results indicate that the increasing electrolyte pH leads to an increase in the near‐surface concentration of cations which positively affects the HER kinetics on the RHE scale. Remarkably, the near saturation effects are also captured for the reaction order of HER in the bulk electrolyte pH, as shown in Figures 5 a and b where the reaction order on the bulk pH decreases from around 0.2 to 0 in going from pH 7 to pH 14. Similar trends in the experimental reaction orders for the cation concentration and the electrolyte pH suggest that these two parameters are tuning the same active species at the interface.

### Probing the Au Interface in Alkaline Media

**Figure 6:**
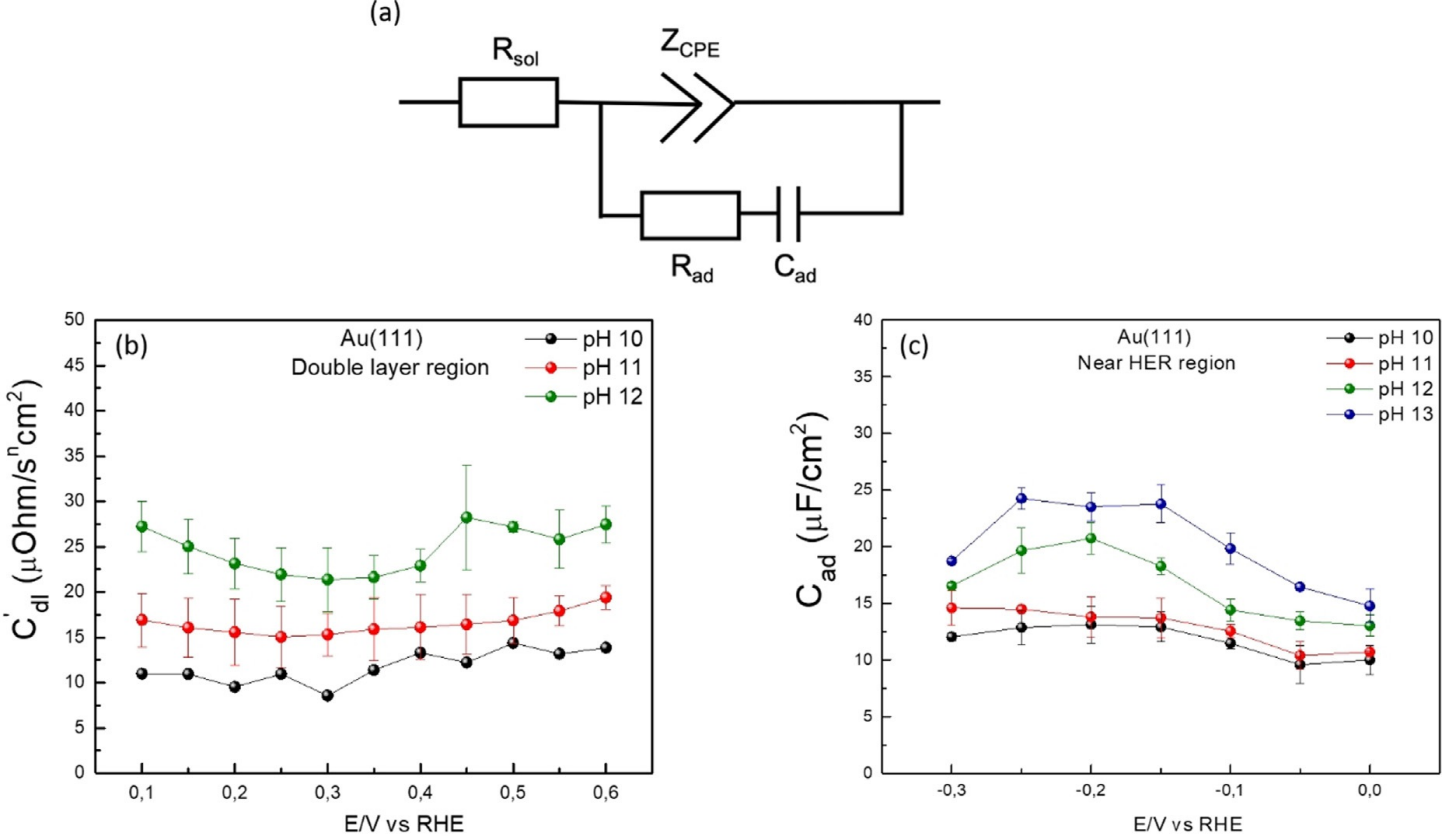
Electrochemical impedance spectroscopy on Au(111) in 0.1 M solutions at different pH values. a) The equivalent electrical circuit (EEC) that was used to fit the data, featuring the *R*_sol_ term for the internal solution resistance, constant phase element term (*Z*_CPE_) which is used to derive the double layer capacitance (*Z*_CPE_=*C*′_dl_^−1^(*jω*)^−*n*^) under the assumption that *C*′_dl_ represents the true double layer capacitance (*C*_dl_) in the limit of *n*≥0.95 and *R*_ad_, *C*_ad_ terms for the charge transfer resistance and the capacitance related to any adsorption phenomena at the interface. In the double layer region, where no Faradaic adsorption processes happen, *R*_ad_ and *C*_ad_ terms can be neglected as the main contribution to the overall capacitance comes from the *Z*_CPE_ (*C*′_dl_) term, whereas near the onset of HER it is assumed that the main contribution to the overall capacitance comes from the *C*_ad_ term as the exponent term (*n*) for *Z*_CPE_ becomes quite low (*n*≈0.2), thereby losing any physical meaning. We plot the specific capacitance (μF cm^−2^) as obtained through these fits in b) double layer region and in c) near‐HER region, given by the *C*_dl_ term and *C*_ad_ term, respectively, as a function of the applied potential (vs. RHE).

In order to gain a better understanding of the pH dependence of the Au‐water interface in alkaline media, we performed electrochemical impedance spectroscopy (EIS) to determine the capacitance of the Au(111) electrode‐electrolyte interface at different pH values. Figure 6 summarizes these results, where we fit the EIS data with the circuit shown in Figure 6 a and in Figure 6 b and c we plot the specific capacitance as obtained through these fits as a function of the applied potential (vs. RHE) in the double layer region and the near HER region, respectively. It should be noted here that in order to fit the double‐layer capacitance (*C*_dl_) we have to employ a constant phase element (CPE; *Z*_CPE_=*C*′_dl_^−1^ (*jω*)^−*n*^) indicating that the double layer behaves non‐ideally in these experiments, possibly due to interfacial heterogeneities arising from the surface disorder or due to the surface position dependent ion adsorption/diffusion phenomena.^[14]^ Interestingly, even with the Au(111) electrode, the CPE exponent term (*n*) decreases with the increasing pH (see Figure S10 in the Supporting Information), indicating that the CPE behavior of the double layer must originate from the changes in the metal‐electrolyte interactions as the electrolyte pH is changed. In fact, Lipkowski and co‐workers have shown using in situ infrared spectroscopy measurements that anions such as OH^−^, SO_4_^2−^ and Cl^−^ can adsorb on Au(111) surface in the double layer region under near‐neutral and alkaline conditions.^[15]^ Thus, it can be expected that in our experiments, OH^−^ specific adsorption at the interface contributes to pseudocapacitive charging resulting in the CPE behavior of the double layer. Consequently, it is impossible to differentiate between the physical meaning of the double‐layer capacitance (*C*_dl_) term and the adsorption capacitance (*C*_ad_) term in the EEC (shown in Figure 6 a) of the system, since both of these terms represent changes in the ion adsorption behavior at the interface.

**Figure 7:**
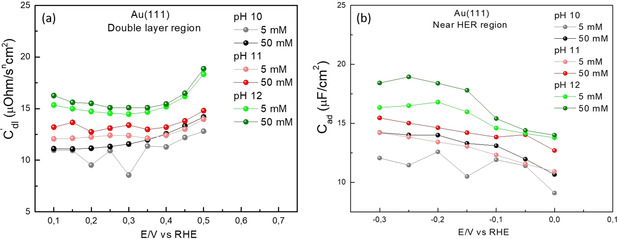
Electrochemical impedance spectroscopy on Au(111) at different cation concentrations (and pH) in the electrolyte, where the capacitance is derived from the same EEC as in Figure 6 a. Here we plot the specific capacitance (μF cm^−2^) as obtained from the fits at pH 10, pH 11, and pH 12, in the a) double layer region (*C*_dl_) and b) near HER region (*C*_ad_) at two different concentrations of the NaClO_4_, namely, 5 mM and 50 mM, represented by light and dark data points, respectively.

Additionally, it should be noted that all the potentials applied during the impedance measurements are more negative than the *E*_pzc_ of Au(111) (0.474 V vs. RHE at pH 0; *E*_pzc_=*E* ^o^_pzc_ + 0.059 pH),^[13c]^ resulting in a net negative interfacial electric field (Δ*E*=*E*−*E*_pzc_) at the electrode at all the investigated potentials. Hence, an increase in the interfacial capacitance (both *C*_dl_ and *C*_ad_) with the increasing pH suggests a corresponding increase in the interfacial concentration of the cations. In order to confirm this effect, we performed additional EIS measurements at a constant pH with varying concentration of the cations in the electrolyte (shown in Figure 7). In agreement with our hypothesis, we observe an increase in the interfacial capacitance with the increasing cation concentration in the electrolyte. These analogous variations in the capacitance curves evidence that these two parameters, namely, the electrolyte pH and the bulk cation concentration, affect the electrode‐electrolyte interface in a similar manner.

**Figure 8:**
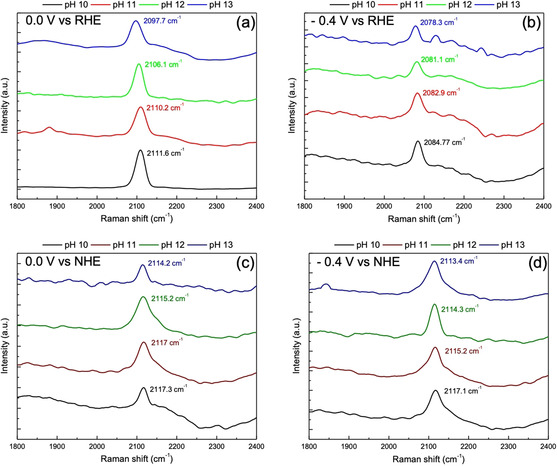
In situ surface Raman spectra of hydrogen adsorption on a roughened Au polycrystalline electrode at a) 0.0 V, b) −0.4 V vs. RHE and c) 0.0 V, d) −0.4 V vs. NHE obtained in 0.1 M NaOH (pH 13), 0.01 M NaOH +0.09 M NaClO_4_ (pH 12), 0.001 M NaOH + 0.099 M NaClO_4_ (pH 11), and 0.0001 M NaOH +0.0999 M NaClO_4_ (pH 10), in Ar‐saturated environment.

In order to gain further insights into the intrinsic pH dependence of the Au‐water interface during hydrogen evolution we also performed in situ surface enhanced Raman spectroscopy (SERS) to probe the changes in the Au‐H vibrational band as a function of the electrolyte pH. Figure 8 presents the SERS results, where we attribute the band located around 2100 cm^−1^ to the H bonded on top of the Au surface atom.^[16]^ These results show that the Au‐H vibrational band shifts to lower wavenumbers with the increasing electrolyte pH (pH 10 to pH 13) indicating that the nature of the adsorbed hydrogen is indeed pH dependent. Moreover, the pH dependent shift in the band occurs both on the RHE scale and on the NHE scale, suggesting that these changes have an intrinsic pH dependence and they are not convoluted by the changes in the near‐surface cation concentration. In principle, a shift to a lower wavenumbers with the increasing pH could indicate that the hydrogen bond strength decreases with the increasing pH, though one must be careful in electro‐sorption systems to correlate changes in metal‐adsorbate frequencies to corresponding changes in binding energies because there is no theoretical basis for such a correlation.^[17]^ Interestingly, Mao and co‐workers have previously observed a similar pH dependence for the Pt‐H vibrational band.^[16c]^ Notably, the observed Stark tuning effect for the Au‐H band (see Supporting Information Figure S12) is also similar to the previously reported Stark tuning effect for the Pt‐H band.^[16c]^

While these spectroscopy measurements do not allow us to draw detailed quantitative conclusions, together with the impedance data, they do lead to two important qualitative conclusions that are in line with the conclusions of the previous section: (i) there is an intrinsic pH dependence of the (double layer structure of the) gold‐aqueous electrolyte interface, presumably resulting in associated changes in the (weak) specific OH^−^ adsorption in double layer and the strength of the hydrogen adsorption in the HER window, and (ii) cation and pH effects are convoluted in the sense that higher pH invoke higher near‐surface cation concentrations. We believe that an investigation into the nature of conclusion (i) requires a detailed study of its own.

## Discussion

It is now well established that in alkaline media, the HER kinetics cannot be described aptly by only taking the changes in the HBE into account. There is ample experimental proof that in the alkaline pH window traditional descriptors fail to capture the complex non‐Nernstian pH dependence of HER activity.^[18]^ Instead, various groups have successfully identified alternative interfacial parameters that impact the HER kinetics in alkaline media, for example the potential of zero charge (or the interfacial electric field strength), the binding energy of the co‐adsorbed hydroxyl ion or oxophylicity of surface sites in general, and the solvation energy of the spectator cations.[^6a^, ^8^, ^11^, ^19^] These parameters are believed to influence the rate‐determining step in alkaline media, that is, H_2_O + e^−^ + * → H−* + OH^−^. However, in order to arrive at a unified theory that can capture all the experimental anomalies that exist in present literature, it is important to understand how these key parameters influence each other and which reaction conditions can be realized to amplify their effect on the HER kinetics.

Our results show that on Au electrodes, the overall activity for HER in alkaline media is indeed controlled the first electron transfer step, hence by the barrier of the electrochemical water dissociation (H_2_O + e^−^ + * → H−* + OH^−^). This rate of this reaction is enhanced in the presence of cations near the surface under moderately alkaline conditions (pH 11). We note here that the cation concentration dependence of HER is reminiscent of the studies that have been conducted by Markovic and co‐workers on modified transition metal electrodes.[^6a^, ^6b^, ^6c^] Moreover, based on their experimental findings, they proposed a bi‐functional mechanism for HER where in addition to the H adsorption, the HER activity is also dependent on the OH adsorption at the interface. However, in accordance with the recent studies by Tang and co‐workers, we believe that any direct involvement of the adsorbed OH species in the HER mechanism would not be expected.^[20]^ It is more likely that the cations improve the intrinsic kinetics of HER by bringing down the kinetic barrier for the electrochemical water dissociation step. This is very similar to a model suggested recently by our group in which the hydroxide is (transiently) stabilized by an oxophilic adatom on the platinum electrode.^[19]^ Therefore, the reactivity scales with (theoretical) oxophilicity of the adatoms, even though under conditions of HER, no OH is (or is expected to be) adsorbed at the interface, as supported by first‐principles density functional theory calculations. Hence, we propose here that the cations in the (outer‐)Helmholtz plane promote the hydrogen evolution by likewise favorably interacting with the transition state of the reaction (H_2_O + e^−^ + * + cat^+^ → *H – OH^δ−^–cat^+^ + (1−δ)e^−^ → *H + OH^−^ + cat^+^) thereby increasing the probability of electrochemical water dissociation at the metal interface.

An alternative explanation would invoke the idea that the electric field in the double layer is affected by the cation concentration. We have previously argued that this electric field effect may influence the reorganization of interfacial water and thereby the rate of OH^−^ transfer through the double layer. We advocate here the model that cations favorably interact locally with the negatively‐charged transition state because it is a simple and intuitive idea, but as in our recent paper,^[19]^ we cannot fully discard the more “global” electric field model (generating global field lines normal to the electrode surface). We note that this local promoting effect of cations (generating electric field radiating from the ion), stabilizing a key intermediate, has also been suggested for the electrocatalytic CO_2_ reduction.^[21]^ However, a more global effect has also been suggested, by some of the same authors.^[22]^

At increasingly negative potential, we expect the concentration of cations near the surface to level off and to eventually to reach a maximum (very much like in a Langmuir or Frumkin isotherm). This would explain the near‐zero reaction order in cation concentration at pH 12 (as shown in Figure 2) and, at very high cation concentration, the observed negative reaction order. The negative reaction order suggests an inhibitive effect, which is traditionally modeled by site blocking. Since it is unclear whether the cations actually chemically adsorb on surface sites (but see the computational work by Janik and co‐workers)^[23]^ or rather accumulate in double layer, the exact origin of this inhibition may not be fully clear. We do expect however that a high accumulation of cations in the double layer may have an adverse effect on the extent to which reactive water can reach the gold surface.

Therefore, an “empirical” rate law accounting for the observed cation effects would take the form:

(1) $$v_{1}=\;k_{1}^{eff,0}\left(1-\frac{\Gamma_{cat,s}}{\Gamma_{max}}\right)exp\left(-\frac{\alpha FE}{RT}\right){\;\Gamma }_{cat,s}^{\gamma }$$

$k_{1}^{eff,0}$ is some effective standard rate constant, *α* is the transfer coefficient, *F* is Faraday's constant (96485 C mol^−1^), *E* is the applied potential with respect to the standard potential of the reaction, *R* is the universal gas constant (8.314 J K^−1^ mol^−1^), *T* is the temperature (K), Γ_cat,s_ is the surface concentration of cations (in mol cm^−2^), Γ_max_ is the maximum (saturated) surface concentration of cations, and γ is the (empirical) reaction order in the (local) cation concentration. This expression could be rewritten to show more explicitly that the activation energy of the reaction is lowered by a factor γRTln (Γ_cat,s_/Γ_max_) due to the presence of cations near the interface. The potential dependence of Γ_cat,s_ is then given by its corresponding isotherm expression (the Frumkin isotherm probably being the simplest reasonable candidate):

(2) $$\frac{\Gamma_{cat,s}}{\Gamma_{max}-\Gamma_{cat,s}}=K^{{}^{\circ }}\exp\left(\frac{F\left(E-E_{pzc}\right)}{RT}\right)\exp\left(-g\frac{\Gamma_{cat,s}\;}{\Gamma_{max}\;}\right){\left[{cat}^{+}\right]}_{b}$$

where $K^{{}^{\circ }}$ is the standard equilibrium constant for cation adsorption at the bare surface, *E*_pzc_ is the potential of zero charge (*E*_pzc_=*E* ^o^_pzc_ + 0.059 pH_surface_ vs. RHE) which incorporates the pH dependence of the HER kinetics, Γ_max_ is the maximum (saturated) surface concentration of cations, *g* is the Frumkin interaction parameter (*g*>0 signifying repulsive interactions), and [cat^+^]_b_ is the bulk cation concentration. We stress that Equations (1) and (2) are not supposed to model our data quantitatively; they are only meant to illustrate the various interrelated effects of cations in a simple model.

Alternatively, it can also be argued that the buffering effect of Na^+^ cations due to their hydrolysis at more alkaline conditions (pK_hydrolysis_=14.2) can lower the OH^−^ surface concentration [OH^−^]_s_ as the bulk electrolyte pH becomes more alkaline, thus countering the increase in the Γ_cat,s_.^[21]^ However, the experimentally (and theoretically) observed drop in the electrolyte pH with the increasing cation concentration (at pH 13; see Figure S13 in the Supporting Information) is small in comparison with the observed drop in the HER activity. Hence, it is much more likely that high cation coverage impedes the HER kinetics due to blockage effects.

Interestingly, the pH dependence of the observed reaction orders in cation concentration suggests that in addition to the bulk cation concentration, Γ_cat,s_ can also be tuned via the electrolyte pH. Essentially, we observe that increasing electrolyte pH leads to a corresponding increase in the near surface cation concentration, at a constant potential on the RHE scale, and reaches saturation at pH 12. These results can be reconciled with the previously reported pH dependence of the interfacial electric field (Δ*E*=*E*−*E*_pzc_) due to the positive shift in the *E*_pzc_ with the increasing electrolyte pH (*E*_pzc_=*E* ^o^_pzc_ + 0.059 pH_surface_ vs. RHE)[^8^, ^13a^, ^13b^] which will in‐turn lead to an increase in the Γ_cat,s_ [see Eq. (2)]. An important consequence in terms of Equation (1) and (2) is that it may reproduce the pH dependence of HER on the RHE scale as an implicit function of the cation dependence of HER kinetics. Experimental results shown in the previous section confirm this prediction as we observe an increase in the HER activity on Au with the increasing pH (on the RHE scale; refer to Figure 3). This is interesting because previously, the pH dependence of the local field strength has been correlated rather to the changes in the local solvent structure.[^8^, ^24^] Here we emphasize that any changes in the interfacial electric field will also affect the local composition of the electrolyte thus establishing a direct correlation between the pH dependence and the cation dependence of HER activity. The combined effect of the cations/interfacial electric field on the kinetics of HER will be hard to decouple in an experiment, since they essentially emphasize local vs. global electric field effects that may be difficult to unequivocally separate.

It should be noted here, that on a pH independent scale (NHE or Ag/AgCl) where the *E*_pzc_ does not change with the electrolyte pH (*E*_pzc_=*E* ^o^_pzc_), Equation (1) predicts a pH independent Γ_cat,s_ and hence a pH independent reaction rate at a fixed potential (on the NHE scale). This agrees with the expectation that for a rate‐limiting electrochemical water dissociation step an inherent pH dependence should not exist since no protons or hydroxide ions are involved in the reactant side of the rate limiting reaction (H_2_O + e^−^ + * → H−* + OH^−^). However, the experimental results on the NHE scale show that the HER kinetics become more sluggish with the increasing pH (Figure 4), suggesting that the electrolyte pH has an intrinsic effect on the HER kinetics. These results show that in addition to its subsidiary role in tuning the near‐surface cation concentration on the RHE scale, the electrolyte pH also affects the HER kinetics directly. One possible reason for the intrinsic pH dependence of the HER kinetics could be the changes in the HBE with the changing electrolyte pH. In fact, the pH dependence of the Au‐H vibrational band, as shown in Figure 8, suggests that the nature of the adsorbed hydrogen changes with the electrolyte pH. However, further experimental and theoretical work is required in order to completely understand the intrinsic pH dependence of the HER kinetics on Au in the alkaline media. Importantly, of these two effects, the role of electrolyte pH in tuning Γ_cat,s_ is dominant as it dictates the overall activity trend on the RHE scale where both effects of electrolyte pH should be operational. Additionally, the existence of these two opposing effects also explains the slightly lower reaction orders in the bulk electrolyte pH (≈0.2; pH 7 to pH 12) for a constant cation concentration (0.1 M) in comparison with the reaction orders obtained in cation concentration at moderately alkaline pH (≈0.5 at pH 11).

## Conclusion

From the experimental evidence presented in this paper we conclude that there exists an intricate interrelation between the cation and the pH effects on the HER kinetics in the alkaline media. We have shown here that on Au electrodes, the rate for the sluggish Volmer step is increased when the near‐surface cation concentration is increased. Based on these results, we propose that the cations tune the kinetic energy barrier for HER by favorably interacting with the transition state of the rate determining step (*H – OH^δ−^–cat^+^). Moreover, with the help of kinetic measurements and interfacial capacity measurements we elucidate the indirect role of bulk pH in tuning the near‐surface cation concentration and shed light on the convoluted nature of pH effects for HER activity. Essentially, we observe that on the RHE scale increasing pH results in increasing near‐surface cation concentration, thereby improving the HER kinetics. Interestingly, it appears that in addition to these effects, electrolyte pH also affects the HER kinetics directly, by tuning the pre‐exponential factor of the reaction. However, the overall activity trends demonstrate that near‐surface cation concentration is the more important parameter in describing the HER activity in alkaline media on Au electrodes.

Furthermore, we show that on the Au surface cations provide optimal enhancement of the HER activity at intermediate pH values and that this promotional effect slows down at very high near‐surface cation concentrations (with increasing pH and increasing bulk cation concentration), even becoming inhibitive above a threshold concentration. This suggests that the optimal concentration of cations for promoting HER varies depending on the degree of stabilization required by the transition state of the reaction indicating that the observed activity trends are strongly dependent on the strength of the metal‐water‐cation interactions. Therefore, in order to optimize the activity of HER on different electrocatalysts it is imperative to study these interfaces individually.

To conclude, this work has elucidated the convoluted role of interfacial field strength and the electrolyte cation concentration in tuning the rate of the alkaline Volmer step. Essentially, we consider both the bulk cation concentration and the electrolyte pH affect the local surface concentration of cations, the latter by influencing the local field strength. Interfacial cations alter the kinetic barrier of the water dissociation step by transiently stabilizing the transition state in which the hydroxyl ion splits off from the water molecule. Moreover, the electrolyte pH also affects the nature of the adsorbed hydrogen at the interface, which further indicates a possible pH dependence of the hydrogen bond strength. Our results demonstrate that the different interfacial parameters that are generally proposed to play a key role in the HER kinetics, all play out simultaneously at the gold‐electrolyte interface, thereby making it very difficult to experimentally decouple these effects and identify a single activity descriptor for HER in alkaline media. Instead, different electrode‐electrolyte combinations need to be probed individually in order to assess which parameter is more important for describing the HER activity on a given surface.

## Conflict of interest

The authors declare no conflict of interest.

## Supporting information

As a service to our authors and readers, this journal provides supporting information supplied by the authors. Such materials are peer reviewed and may be re‐organized for online delivery, but are not copy‐edited or typeset. Technical support issues arising from supporting information (other than missing files) should be addressed to the authors.

Supplementary
