# Supplementary material for: The Interrelated Effect of Cations and Electrolyte pH on the Hydrogen Evolution Reaction on Gold Electrodes in Alkaline Media
Source: Angew Chem Int Ed Engl. 2021 May 7;60(24):13452–62. doi: 10.1002/anie.202102803 (PMC8252582; doi:10.1002/anie.202102803)
Supplement: Supplementary file 1 — Supplementary [file ANIE-60-13452-s001.pdf]

Angewandte Chemie
Eine Zeitschrift der Gesellschaft Deutscher Chemiker

Supporting Information

The Interrelated Effect of Cations and Electrolyte pH on the Hydrogen Evolution Reaction on Gold Electrodes in Alkaline Media

*Akansha Goyal and Marc T. M. Koper**

anie_202102803_sm_miscellaneous_information.pdf

Supporting Information

1. Experimental methods

1.1 Chemicals

The electrolytes were prepared from H_2SO_4 (98% by wt. solution, EMSURE, Merck), $HClO_4$ (60% by wt. solution), $NaClO_4$ (99.99%, trace metals basis, Sigma-Aldrich), NaOH (32% by wt. solution, analysis grade, Merck) and Ultrapure water (MilliQ gradient, ≥18.2 MΩcm, TOC < 5 ppb). Ar (6.0 purity, Linde) and H_2 (5.0 purity, Linde) were used for purging the electrolytes. To prepare the roughened Au electrodes for *in situ* Raman studies, $KAu(CN)_2$ (99.95 %, trace metals basis, Sigma-Aldrich), $KAg(CN)_2$ (Sigma-Aldrich), Na_2CO_3 (≥ 99.5%, Sigma-Aldrich) and $HClO_4$ (60% by wt. solution, EMSURE, Merck) were used.

1.2 General electrochemical methods

The electrochemical measurements at pH 10 to pH 12 were carried out in home-made borosilicate glass cells and measurements at pH 13 were carried out in a home-made PTFE cell. The reference electrode was separated from the working compartment with the help of a Luggin capillary and the counter electrode was a Au wire (99.99% purity), unless otherwise stated. The glassware was cleaned prior to each experiment by boiling it five times in ultrapure water. When not in use, the glassware was stored in 1 g/L solution of $KMnO_4$ (acidified). Before boiling, any traces of $KMnO_4$ and MnO_2 were removed from the glassware by submerging it in a diluted solution of acidified H_2O_2 (few drops of conc. H_2SO_4 and 10-15 mL H_2O_2 in excess water) for half an hour. Before every experiment, the electrolytes were purged for ca. 20 minutes with Ar to remove any dissolved oxygen form the electrolyte. Moreover, during the measurement, Ar was also bubbled over the headspace of the electrochemical cell, in order to eliminate any interference from the ambient oxygen. A home-made reversible hydrogen electrode (RHE) or a Ag/AgCl reference (Pine research instrumentation, sat. NaCl, E = 0.197 V vs standard hydrogen electrode) were used as the reference electrode in all the experiments. All the electrochemical measurements were carried out using a Biologic (VSP-300) potentiostat and a MSR rotator (Pine Research). For all the CVs taken, 85% Ohmic drop compensation was performed and for all the steady-state potentiostatic measurements 100% Ohmic drop compensation was applied. The Ohmic drop of the electrolyte was determined by carrying out electrochemical impedance spectroscopy

(EIS) at 0.05 V (vs RHE; double-layer region). All the measurements with Au polycrystalline were performed with E6/E5 ChangeDisk tips in a PEEK shroud (Pine Research) and all the measurements with Au(111) were performed with a home-made PEEK shroud compatible with the MSR rotator.
For the pre-electrolysis studies, a home-made PTFE cell was used in which overnight (16 hrs.) chronopotentiometry was performed in 0.1 M NaOH electrolyte at a constant current of -1 mA and polycrystalline Pt wires were used, both as the counter and as the working electrode. Thereafter, the electrodes were removed from the electrolyte under potential control. The results from the pre-electrolysis studies are shown in Figure S7 (Supporting Information).

1.3 Working electrode preparation

Au Polycrystalline: Before each experiment, the Au polycrystalline disk (diameter = 5mm, Pine instruments) was mechanically polished on Buehler micro-polishing cloth (8 inches) with decreasing sizes of diamond polishing suspension, namely, 3µm, 1 µm and 0.25µm. Next, the disk was sonicated in ultrapure water and acetone for 10 minutes to remove any organic/inorganic impurities, and mounted on the RDE tip. Thereafter, the Au polycrystalline disk was electrochemically polished in 0.1 M H_2SO_4 (0.05 V to 1.75 V vs RHE, 200 cycles at a scan rate of 1 V s^{-1}) by going to the Au oxide formation and reduction region.[1] Prior to each experiment, a characterization cyclic voltammetry (CV) of the disk was obtained in the same potential window where the electrochemical polishing was performed (at a scan rate of 50 mV s^{-1}), as shown in Figure S1 (Supporting Information). For calculating the current densities in the HER activity the electrochemically active surface area (ECSA) of the disk was determined by calculating the charge from the reduction peak for the Au oxide in the characterization CV and dividing it by the specific charge of one monolayer of Au (390 µC cm^{-2}).[1] The working electrode was then used for the electrochemical measurements.
Au(111): Before each experiment, the Au(111) disk (diameter = 7mm) was prepared by performing multiple cycles (≈ 10) of flame annealing (ca. 1 min) and quenching (MilliQ water).[2] Thereafter, it was transferred to the electrochemical cell with a protective layer of MilliQ water. The quality of the single crystal preparation was ascertained by checking its double layer features in 0.1 M H_2SO_4 (0.1 V to 1.15 V vs RHE), as shown in Figure S1 (Supporting Information). For calculating the current densities in the HER activity experiments, the geometric surface area of the crystal (0.38 cm^2) was used.

1.4 Electrochemical measurements for HER activity

Table 1 Compositions of the electrolytes employed for the pH dependence studies of HER

Bulk pH	$[HClO_4]$ / M	[NaOH] /M	$[NaClO_4]$ /M	Total ion concentration /M
1	10^{-1}	—	—	2×10^{-1}
2	10^{-2}	—	9×10^{-2}	2×10^{-1}
3	10^{-3}	—	9.9×10^{-2}	2×10^{-1}
7	—	—	10^{-1}	2×10^{-1}
10	—	10^{-4}	9.99×10^{-2}	2×10^{-1}
11	—	10^{-3}	9.9×10^{-2}	2×10^{-1}
12	—	10^{-2}	9×10^{-2}	2×10^{-1}
13	—	10^{-1}	—	2×10^{-1}
14	—	10^{0}	—	2×10^{0}

All the studies for the pH dependence of HER were done in Ar sat. 0.1 M electrolytes (except pH =14), as shown Table 1. The CVs were taken in the potential window of 0 V to -0.65 V vs RHE at a scan rate of 25 mVs^{-1} and the working electrode was rotated at 2500 rpm in every measurement. Additionally, to obtain the Tafel data, chronoamperometry was performed in the potential window of the CVs at 50 mV potential steps while rotating the electrode (2500 rpm). Generally, 20 seconds per potential step were enough to reach the steady-state. EIS measurements were performed at different pH values (pH 10 to pH 13) with Au(111) in two potential windows, the double layer region (0.1 V to 0.6 V vs RHE) and the near HER region (0 V to -0.3 V vs RHE), with frequencies ranging from 30 KHz to 1 Hz and a peak to peak amplitude of 5 mV. Moreover, a 10 μF shunt capacitor bridge was added between a secondary counter electrode (Au wire) and the reference electrode in order to eliminate any artefacts caused by the non-ideal behavior of the potentiostat at high frequencies. The impedance data was fit with the help of EIS Zfit (part of Biologic's EC-Lab software) by using the equivalent electric circuit shown in Figure 5a. The typical EIS plots (and fits) obtained during the experiments are shown in Figure S9, S10 and S11 (Supporting Information).

Table 2 Compositions of the electrolytes employed for the cation concentration dependence studies of HER

Bulk pH	[NaOH] /M	[$NaClO_4$] /M	Total ion concentration /M
11	10^{-3}	5×10^{-3}	1.2×10^{-2}
		5×10^{-2}	1.02×10^{-1}
		2.5×10^{-1}	5.02×10^{-1}
		5×10^{-1}	$\approx 10^{0}$
		10^{0}	2×10^{0}
12	10^{-2}	5×10^{-3}	3×10^{-2}
		5×10^{-2}	1.2×10^{-1}
		2.5×10^{-1}	5.2×10^{-1}
		5×10^{-1}	1.02×10^{0}
		10^{0}	2.02×10^{0}
13	10^{-1}	5×10^{-3}	2.1×10^{-1}
		5×10^{-2}	3×10^{-1}
		2.5×10^{-1}	7×10^{-1}
		5×10^{-1}	1.2×10^{0}
		10^{0}	2.2×10^{0}

All the studies for the bulk cation concentration dependence of HER were done at pH 11 (10^{-3} M NaOH), pH 12 (10^{-2} M NaOH) and pH 13 (0.1 M NaOH) where the cation concentration was varied in the electrolyte by adjusting the concentration of $NaClO_4$, as shown in Table 2. The CVs were taken in the potential window of 0 V to -0.65 V vs RHE at a scan rate of 25 mVs^{-1} and the working electrode was rotated at 2500 rpm in every measurement. Additionally, to obtain the Tafel data, chronoamperometry was performed in the potential window of the CVs at 50 mV potential steps while rotating the electrode (2500 rpm). Generally, 20 seconds per potential step were enough to reach the steady-state. Impedance measurements were also performed in the double-layer region (0.1 V to 0.6 V vs RHE) and the near HER region (0 V to -0.3 V vs RHE) at 2 different concentrations of $NaClO_4$, namely 5 mM and 50 mM and different

electrolyte pH values (pH 10 to pH 12). The parameters of the EIS measurements were same as above.

1.5 *In situ* surface enhanced Raman Spectroscopy measurements

In situ surface enhanced Raman spectroscopy (SERS) measurements were performed with a LabRam HR800 (Horiba Jobin Yvon) confocal microscope, which featured a HeNe laser (632.81 nm) where the laser beam was introduced through an objective lens to the roughened Au electrode in a vertical arrangement.[3] In order to avoid beam damage the laser beam was attenuated by a factor of 10 resulting in a measured beam energy at the location of the Raman cell of 0.28 W. The measurements were performed with electrolytes of different pH (pH 10 to pH 13) with a fixed total ion concentration (0.2 M; refer to Table 1) where the spectra were recorded in the potential window of 0.2 V to -0.6 V (vs RHE and vs NHE) at 200 mV potential steps. It was not possible to go more negative than -0.6 V (vs RHE) because beyond these potentials, the hydrogen bubbles attached to the surface disturbed the vertical spatial resolution in the optical window. The polycrystalline Au electrode was prepared for the SERS studies by electrochemically depositing roughened nano-porous Au channels at the flat Au electrode by following the procedure outlined by Searson and co-workers and Eich and co-workers.[4] Briefly, a binary alloy solution of $Ag_{80}Au_{20}$ was prepared with $KAg(CN)_2$ and $KAu(CN)_2$ in 0.25 M Na_2CO_3 buffer solution and it was electrochemically deposited onto the Au substrate electrode at -1.2 V (vs Ag/AgCl) for 7 min. Thereafter, Ag was de-alloyed from the film by electrochemically cycling (20 cycles) the electrode in a 1 M $HClO_4$ solution between 1.05 V to 1.2 V (vs Ag/AgCl) at 5mVs^{-1}. Thereafter, the electrode was ready for the SERS measurements.

2. Supplementary Figures

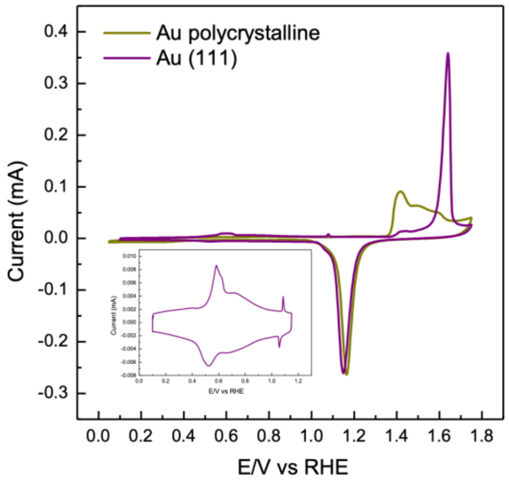

Figure S1 Characteristic cyclic voltammograms for Au polycrystalline surface (golden) and Au(111) surface (purple) obtained in 0.1 M H_2SO_4 in Ar sat. environment at 50 mVs^{-1}. The inset shows the characteristic double-layer features of the Au(111) surface in the potential window of 0.1 V to 1.15 V (vs RHE).

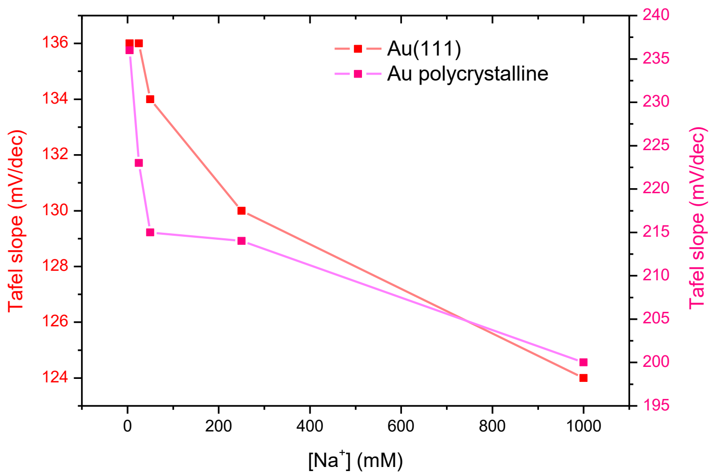

Figure S2 Tafel slopes obtained for Au polycrystalline surface (pink; right hand side on the y-axis) and Au(111) surface (red; left hand side on the y-axis) at pH 11 for different Na $^{+}$cation concentration in the electrolyte (plotted on the x-axis). The data is obtained from the steady-state currents obtained at 50 mV potential steps in the HER region.

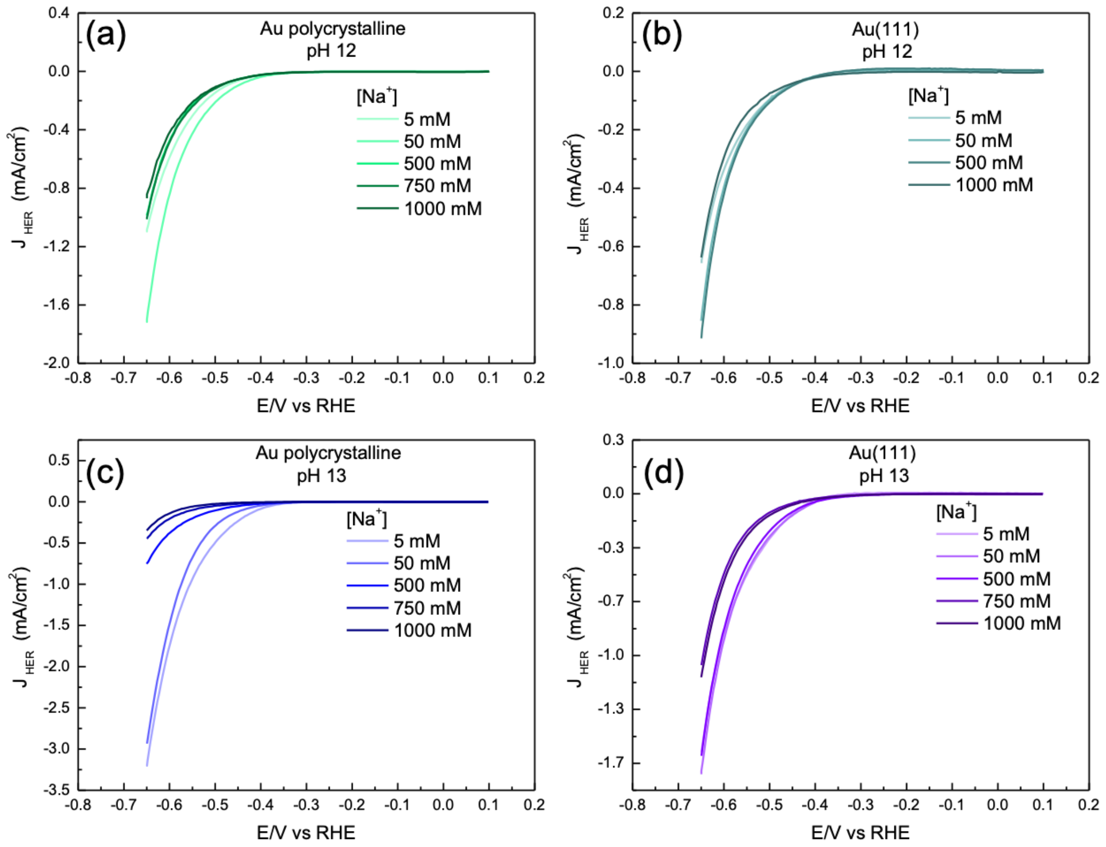

Figure S3 Cyclic voltammograms obtained for HER at pH 12 on (a) Au polycrystalline surface and (b) Au(111) surface and at pH 13 on (c) Au polycrystalline surface and (d) Au(111) surface at 2500 rpm for different concentrations of $NaClO_4$ (5 mM, 50 mM, 500 mM, 750 mM and 1000 mM) in Ar sat. environment at 25 mVs $^{-1}$.

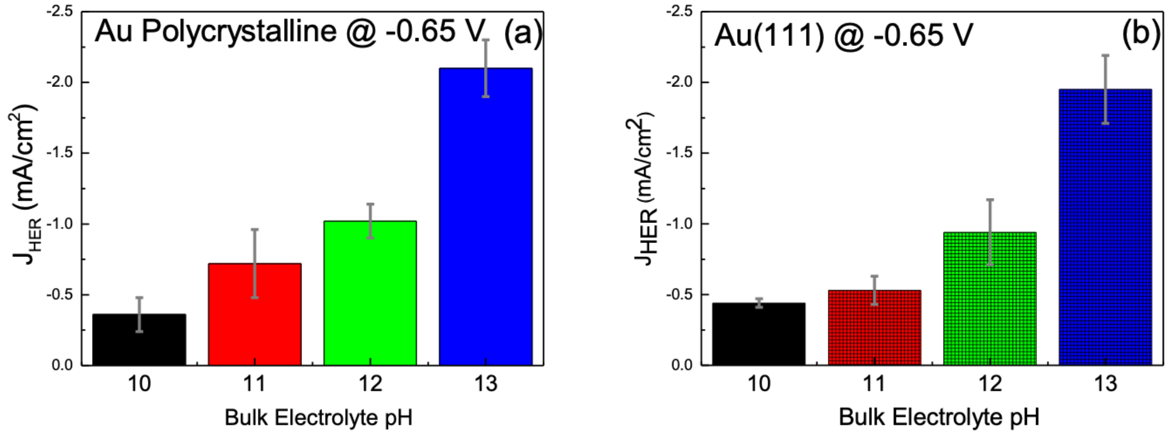

Figure S4 Characteristic current densities obtained for (a) Au polycrystalline surface and (b) Au(111) surface in 0.1 M electrolytes at different bulk pH (pH 10 to pH 13) in Ar sat. environment at - 0.65 V (vs RHE) at 2500 rpm.

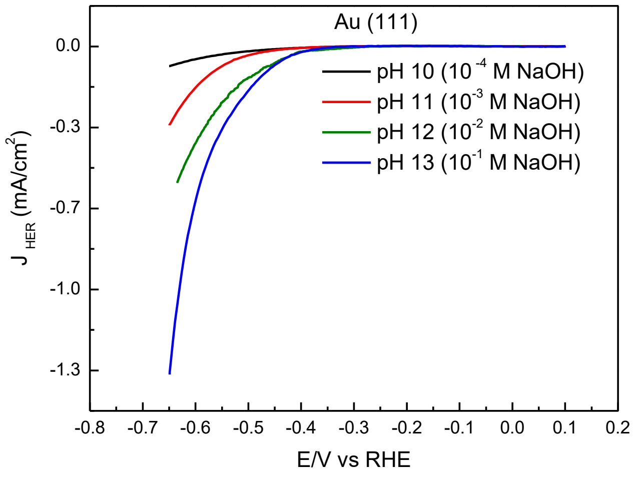

Figure S5 Cyclic voltammograms obtained for HER on Au(111) surface in 0.1 M NaOH (pH 13), 0.01 M NaOH (pH 12) , 0.001 M NaOH (pH 11) and 0.0001 M NaOH (pH 10), in Ar sat. environment at 25 mVs^{-1} under stationary hanging meniscus configuration. In these experiments the ionic strength of the electrolyte is not fixed.

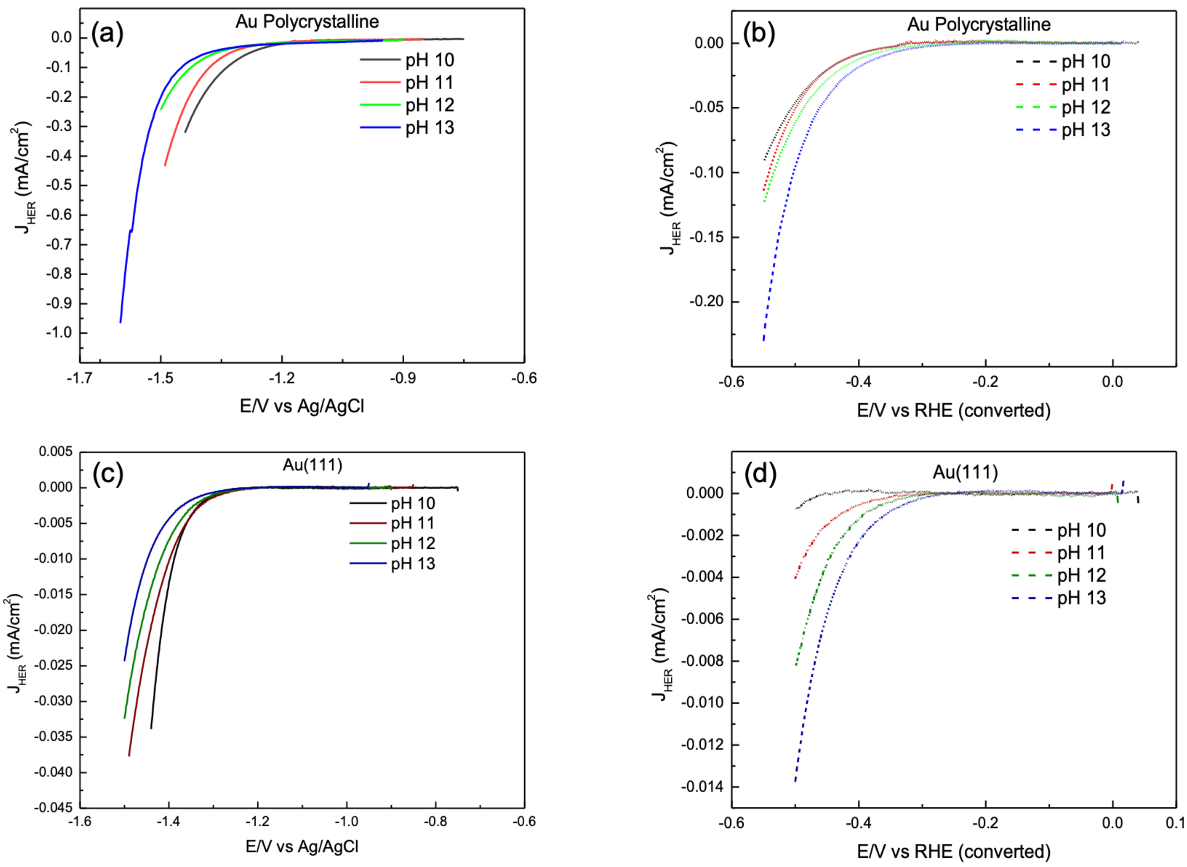

Figure S6 Cyclic voltammograms obtained for HER on (a) Au polycrystalline surface and (c) (d) Au(111) surface at 2500 rpm in 0.1 M NaOH (pH 13), 0.01 M NaOH +0.09 M $NaClO_4$ (pH 12) , 0.001 M NaOH + 0.099 M $NaClO_4$ (pH 11) and 0.0001 M NaOH +0.0999 M $NaClO_4$ (pH 10), in Ar sat. environment at 25 mVs^{-1} with a Ag/AgCl reference and in (b), (d) the data is plotted on the RHE scale where the potentials are converted post experiment from (a) and (c) respectively.

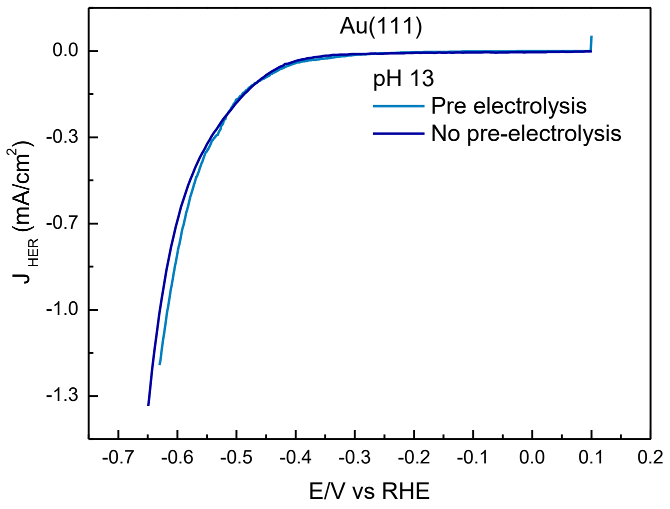

Figure S7 Comparison of the cyclic voltammograms obtained for HER on Au(111) surface in the pre-electrolyzed electrolyte (light blue) and non-pre-electrolyzed electrolyte (dark blue) at pH 13 in Ar sat. environment at 25 mVs^{-1}.

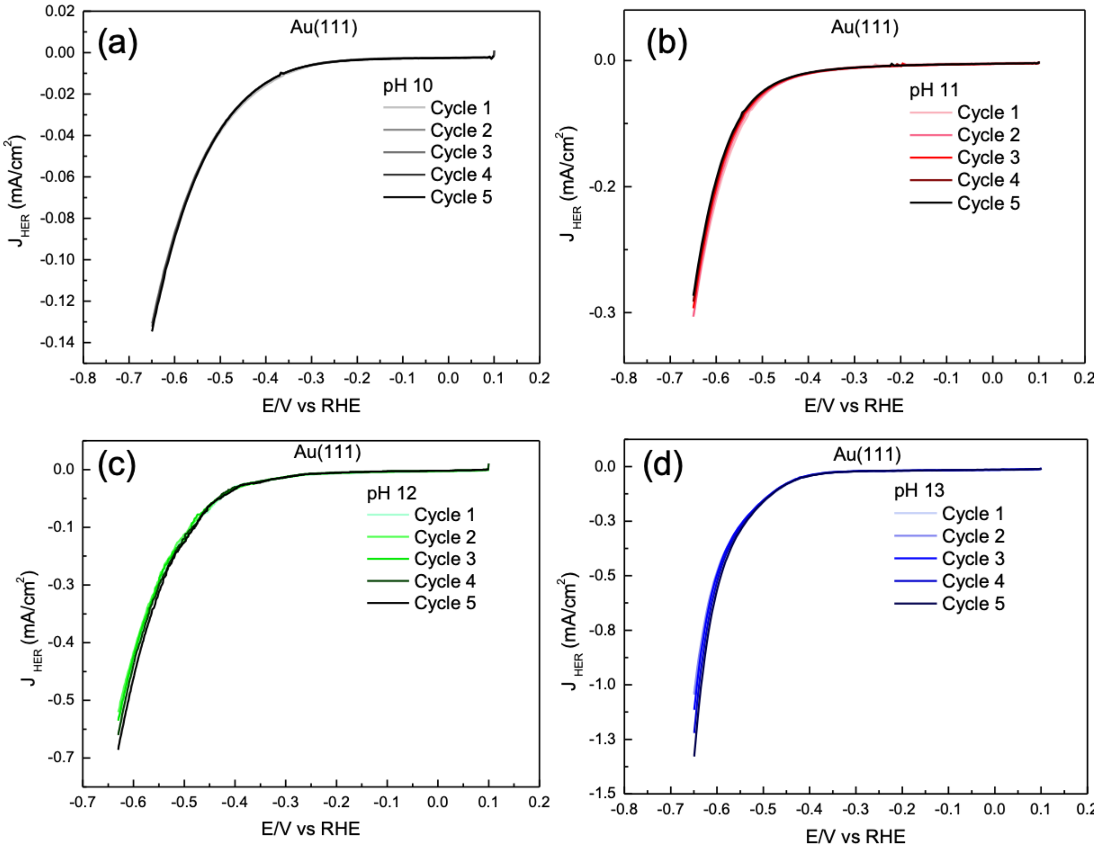

Figure S8 First five cycles of the cyclic voltammograms obtained for HER on Au(111) surface at (a) pH 10, ,(b) pH 11, (c) pH 12 and (d) pH 13 in Ar sat. environment at 25 mVs^{-1}. In all the experiments a stable current density is obtained in the first five subsequent cycles.

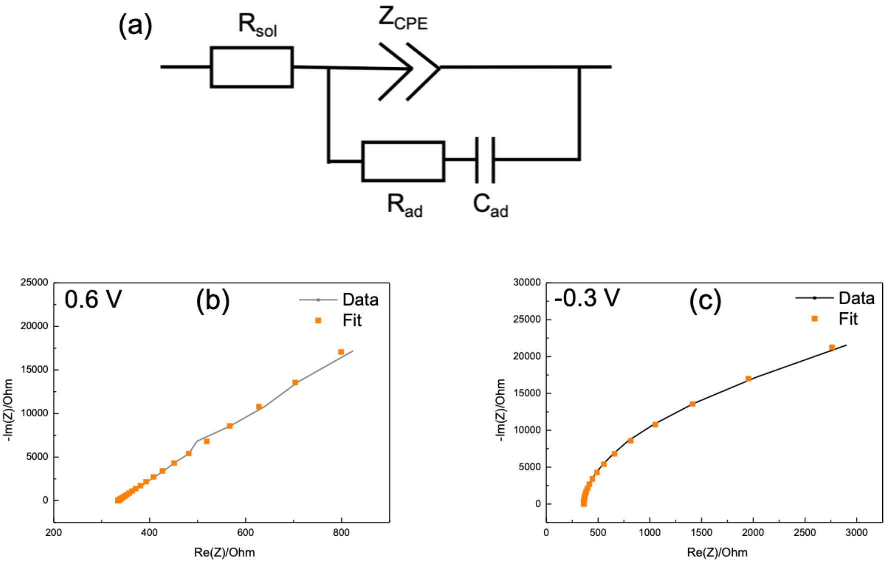

Figure S9 Typical EIS data plotted in the Nyguist impedance representation where the fits are obtained with the equivalent electric circuit (EEC) shown in (a) and the plots obtained at pH 10 in the (b) double layer region (0.6 V) and (c) near HER region (-0.3 V) are shown for comparison. In general, it was observed that in the double layer, where no Faradaic processes happen, R_{ad} and C_{ad} terms could be neglected as the main contribution in the overall capacitance came from the Z_{CPE} ($Z_{CPE} = C'_{dl}{}^{-1}(j\omega)^{-n}$) term whereas near the onset of HER the constant phase (n) for Z_{CPE} became quite low (around 0.5), thereby losing any real physical meaning and the main contribution in the overall capacitance came from the C_{ad} term.

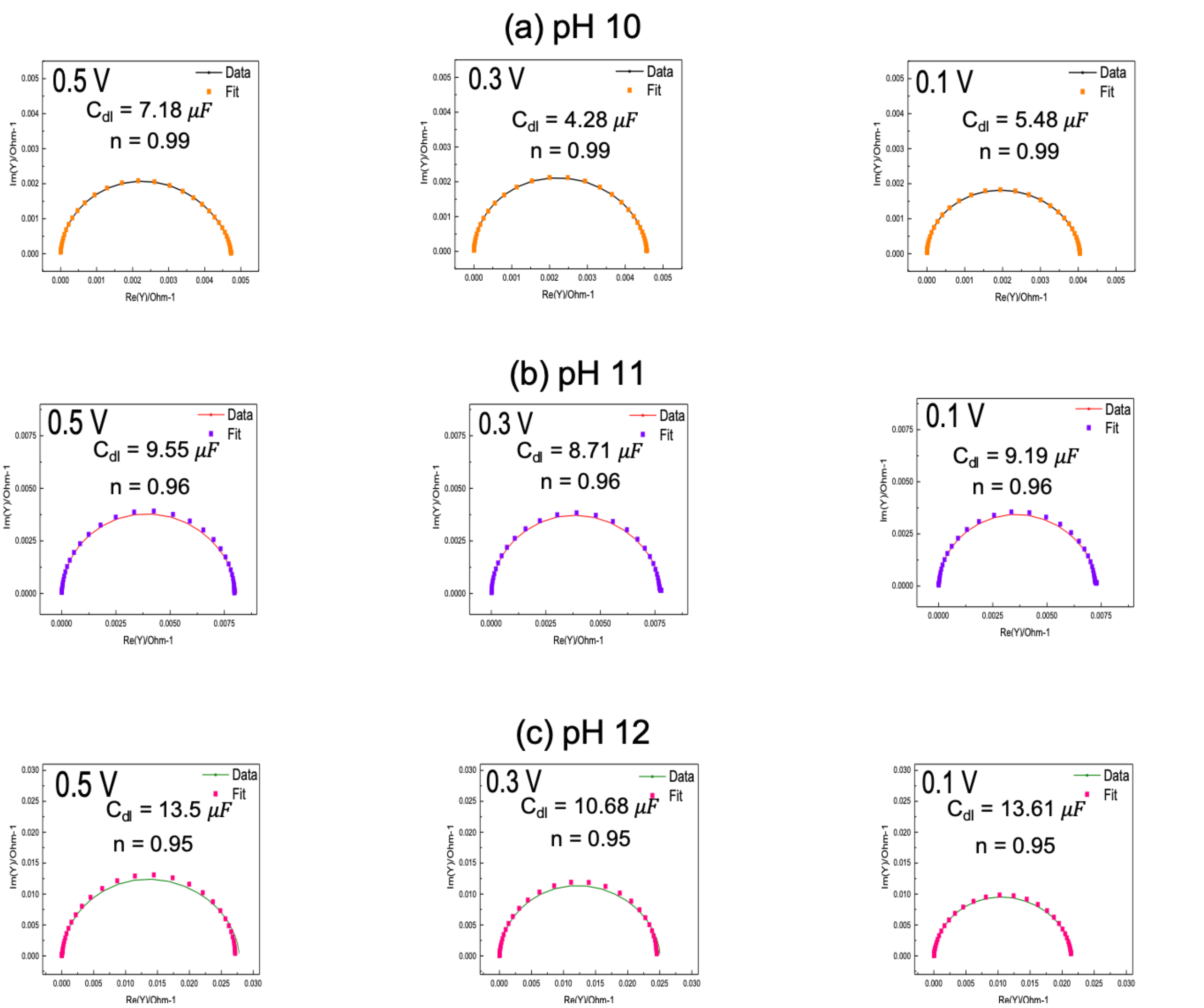

Figure S10 Typical Nyguist admittance plots and the fits obtained from the EIS data in the double layer region (0.5 V, 0.3 V and 0.1 V; left to right) at (a) pH 10, (b) pH 11 and (c) pH 12 where the EEC used to fit the data is the same as shown in Figure S10a. The value of the double layer capacitance (C_{dl}) and the CPE exponent term (n) as obtained from the Z_{CPE} ($Z_{CPE} = C'_{dl}{}^{-1}(j\omega)^{-n}$; it is assumed that C'_{dl} represents the true double layer capacitance (C_{dl}) in the limit of $n \geq 0.95$) term are indicated in the graph where it is observed that the value of C_{dl} increases from pH 10 to pH 12 and the value of n decreases from pH 10 to pH 12. At pH 13 (data not shown) the EEC showed a significant contribution from the C_{ad} term in the double layer such that the exponent term of the double layer capacitance became quite low ($n < 0.9$), thus making it difficult to derive any real physical meaning from the Z_{CPE} term in the double layer.

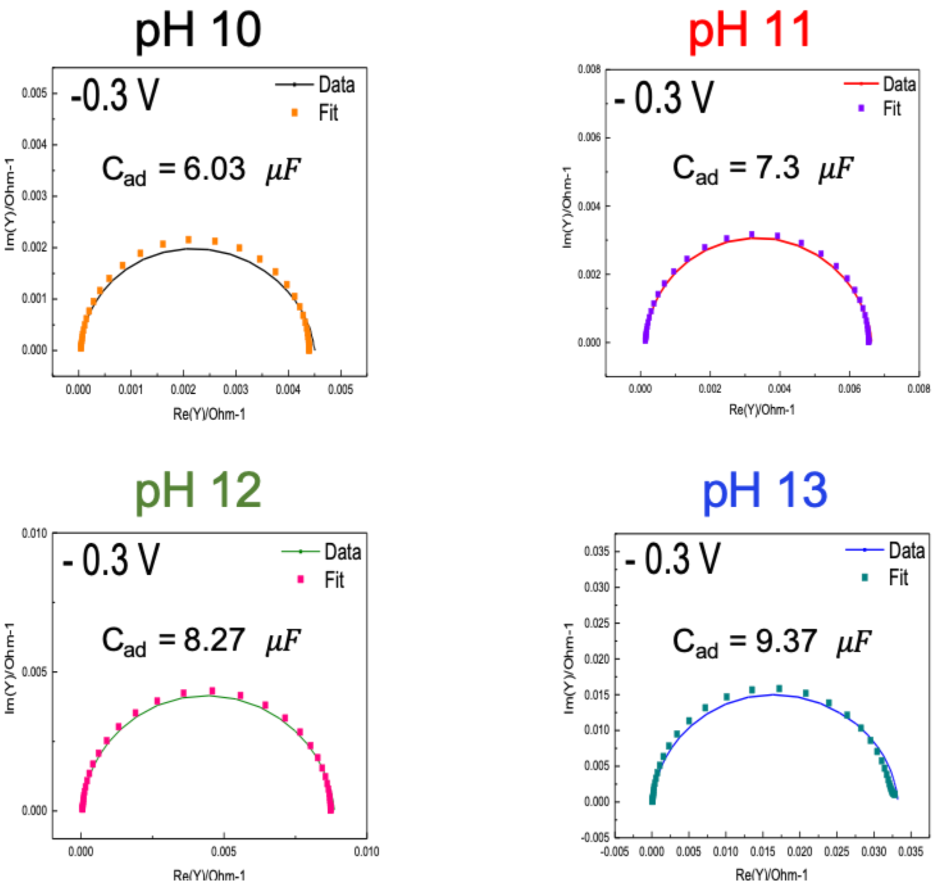

Figure S11 Typical Nyguist admittance plots and the fits obtained from the EIS data in the near HER region (-0.3 V) at pH 10, pH 11, pH 12 and pH 13 where the EEC used to fit the EIS data is same as Figure S11 (a). In the near HER region, the CPE term (Z_{CPE}) representing the double layer capacitance shows very low values for the exponent term (around 0.5) and hence cannot be used to derive any physical meaning. In this region contribution from the C_{ad} (adsorption capacitance) term becomes significant such that the value of C_{ad} increases form pH 10 to pH 13. The value for the C_{ad} obtained through the fits is indicated in the admittance plots.

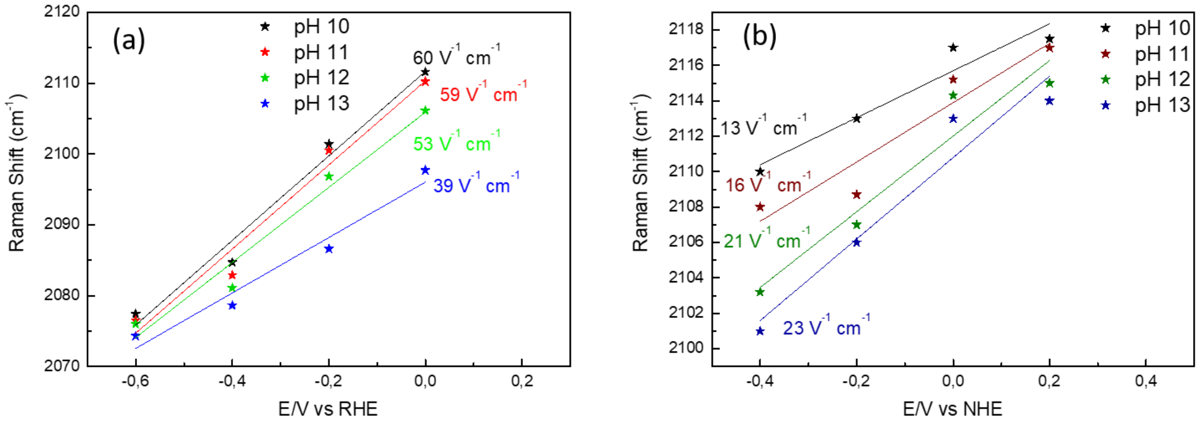

Figure S12 Stark tuning rates (V^{-1} cm^{-1}) obtained from the in situ Surface Raman spectra of hydrogen adsorption on roughened Au polycrystalline electrode on the (a) RHE scale and (b) NHE scale in 0.1 M NaOH (pH 13), 0.01 M NaOH +0.09 M $NaClO_4$ (pH 12) , 0.001 M NaOH + 0.099 M $NaClO_4$ (pH 11) and 0.0001 M NaOH +0.0999 M $NaClO_4$ (pH 10), in Ar sat. environment.

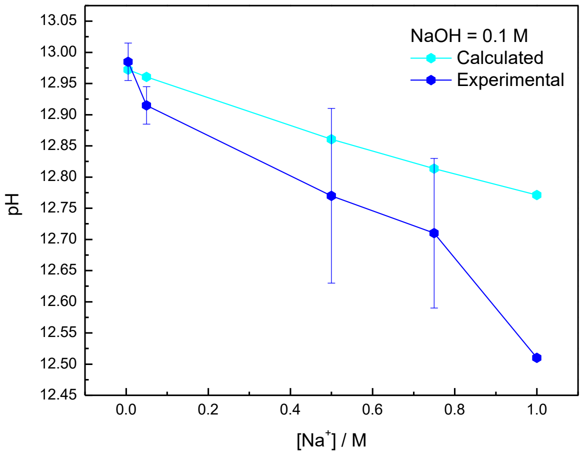

Figure S13 Experimentally measured (blue) and theoretically calculated (cyan) variation in the bulk pH in a 0.1 M NaOH electrolyte with the changing Na^+ cation concentration in the electrolyte (5mM, 50 mM, 500 mM, 750 mM and 1000 mM). For calculating the bulk pH, $pK_{hydrolysis}$ of the Na^+ cation was taken to be 14.2. The discrepancy between the experimentally observed pH and the theoretically calculated pH most likely arises from the changes in the local pH of the electrolyte which are not taken into account in the theoretical calculation of the pH.[5]

References

[1] M. Łukaszewski, M. Soszko, A. Czerwiński, *International Journal of Electrochemical Science* **2016**, *11*, 4442-4469.

[2] N. A. Darwish, P. K. Eggers, W. Yang, M. N. Paddon-Row, J. J. Gooding, in *2010 International Conference on Nanoscience and Nanotechnology*, **2010**, pp. 302-305.

[3] S. C. S. Lai, S. E. F. Kleyn, V. Rosca, M. T. M. Koper, *The Journal of Physical Chemistry C* **2008**, *112*, 19080-19087.

[4] a C. Ji, P. C. Searson, *Applied Physics Letters* **2002**, *81*, 4437-4439; b M. Graf, D. Jalas, J. Weissmüller, A. Y. Petrov, M. Eich, *ACS Catalysis* **2019**, *9*, 3366-3374.

[5] M. R. Singh, Y. Kwon, Y. Lum, J. W. Ager, A. T. Bell, *Journal of the American Chemical Society* **2016**, *138*, 13006-13012.
